# A Practical Treatise on Tropical Dysentery, More Particularly as It Occurs in the East Indies; Illustrated by Cases and Appearances on Dissection: To Which Is Added, a Practical Treatise on Scorbutic Dysentery, with Some Facts and Observations Relative to Scurvy

**Published:** 1819-10-01

**Authors:** 


### XVII.

*A Practical Treatise on Tropical Dysentery, more particularly as it occurs in the East Indies; illustrated by Cases and Appearances on Dissection: to which is added, a Practical Treatise on Scorbutic Dysentery, with some Facts and Observations relative to Scurvy.* By R. W. Bampfield, Esq. Surgeon, Author of an Essay on Hemeralopia, or Night-blindness; and formerly Surgeon of the Belliqueux and Warrior, His Majesty's Ships of the Line, serving in the East and West Indies. Octavo, pp. 352. London, 1819.

An opinion obtains pretty generally amongst the practitioners of this country that tropical diseases are not properly the objects of their study, as they are never likely to fall within the sphere of their actual observation, or to become the objects of their personal responsibility. In consequence, a very considerable degree of supineness is testified towards those works that treat exclusively of warm climates; and few can be prevailed upon to read them with earnestness save the juvenile quota who may chance to be educating for a tropical field of practice. *Haud inexperti loquimur*: we ourselves recollect the apathy with which, in the days of our medical education, we used to listen to the observations about yellow fever, that occasionally fell from the mouths of our instructors, and the nonchalance with which we turned over the leaves of books that described the maladies of the torrid zone. A latent idea seemed to possess our minds that we had "neither part nor lot in the matter," and that a course of reading on such subjects would only be a needless expenditure of time and memory.

So far as we are individually concerned, we have long been taught to feel differently; and all we wish is, that others should become as thoroughly convinced as we are that the notion in question is exceedingly fallacious, short-sighted, and detrimental. The various departments of professional study may be compared to the various organs of the human body, in which the perfection of the whole depends upon the integrity of its several parts. No sooner does a marked improvement take place in one department, than a sympathetic movement takes place, and an animating impulse is communicated to all the rest.

The science of botany has been enlarged by an acquaintance with tropical plants, and no less has the science of medicine been enriched by a knowledge of tropical diseases. Not only have complaints, unknown in temperate climates, been brought within the pale of the nosology, and thus reflected light upon the obscure subject of general pathology, but the intertropical varieties of any disease already familiar, (say fever for instance) have given improved views of its generic character, the nature of its symptoms, and of the curative treatment. In short, it would be easy to show, had we room or leisure for discussion, that the rich store of professional improvement, which is justly the boast of the present æra, has derived some of its most valuable accessions from the spirit and success with which tropical medicine has, of late years, been cultivated.

We have premised these observations for the purpose of awakening the public attention more thoroughly to the literature of tropical diseases in general; and we now proceed to direct that attention to Mr. Bampfield's work in particular. It, in truth, has a just claim to such notice; for it belongs to no ordinary standard of merit. The learning, industry, fidelity of observation, and solidity of judgment which it displays, are extremely creditable to the author; and although, in the exercise of that freedom of thought, which is the legitimate prerogative of every man, we dissent from some of the opinions, both doctrinal and practical, which the book contains, we have no hesitation in bearing testimony to its general excellence.

The work, though there is no formal division of chapters to that effect, may be said to consist of three parts. The 1st treats of *acute*, the 2d of *chronic dysentery*: and the 3d describes another species which the author chooses (whether rightly or not will appear in the sequel) to denominate *scorbutic dysentery*. We shall briefly review each of these in the order here mentioned.

We shall begin by extracting the author's description of the species and varieties he has observed in tropical dysentery, whether acute or chronic.

"Species 1ma. *Dysenteria acuta. Character;* while the fæces are commonly retained, frequent evacuations from the intestines, consisting of mucus, serum, or blood, or a mixture of these take place; and are preceded and attended by pain in some part of the abdomen, and accompanied and followed by tenesmus: pyrexia is not often evident, but is sometimes urgent.

"It varies in degree. (A.) *Dysenteria mitis.* In which the stools are not frequent; the quantity of mucus or serum evacuated is small, and rarely tinged with blood; there is not any fever present; and the pain of the abdomen is never constant, and is only felt, together with tenesmus, about the periods of evacuation.

"(B.) *Dysenteria severa.* In which the stools are frequent, and recur from twelve to forty-eight times, or even oftener, in twenty-four hours; the excretions of mucus, or serum, and the discharges of blood, or a mixture of these three, are copious. The tenesmus and tormina about the periods of evacuation are severely felt; but there is no *constant*, fixed, and acute pain in any part of the abdomen, or unequivocal synocha.

"(C.) *Dysenteria inflammatoria.* In which there is a constant fixed, acute pain of some part of the abdomen or intestinal canal, including the parts contained in the pelvis; unequivocal inflammatory fever (or synocha); obstinate retention of fæces, while there are very frequent and copious dejections of mucus, serum, or blood, or a mixture of these, together with severe tormina and tenesmus. The blood drawn and concreted exhibits the inflammatory buff.

"Species 2da. *Dysenteria chronica.* The acute is frequently succeeded by chronic dysentery, as a sequela of the varieties B and C. In chronic dysentery, the fæces are not retained; but frequent, loose, fæcal stools (a state which, for brevity, I shall term diarrhœa), ensue, mixed with dysenteric excretions, and accompanied with tenesmus and tormina.

"Acute dysentery is sometimes followed by diarrhœa, uncombined with dysenteric excretions, that will be noticed when we come to the treatment."

"Variety (A.) In which diarrhœa is accompanied with an uniform continuance or a frequent recurrence of dysenteric excretions, and of intestinal pains at the periods of evacuation."

"(B.) In which the dysenteric excretions of the intestines are continued and often evacuated, while the bowels observe regular periods of discharging fæces of natural consistence and colour, the same as in health."

"(C.) In which the chronic stage of dysentery is protracted by an ulceration or excoriation of the intestines: the diarrhœa and morbid secretions are maintained; and pus is observed in the evacuations."

"(D.) In which the chronic stage is protracted by a diseased enlargement of the mensteric glands, and, with the following variety, may be considered symptomatic."

"(E.) In which it is maintained by an abscess formed in one of the abdominal viscera or their membranes, and is generally acpanied by hectic fever." P. 2, 3.

This arrangement is certainly logical and luminous, but we scarcely see any advantage in thus splitting down diseases into so many minute varieties. It was the celebrated Cullen who gave currency to this custom; swayed, perhaps, more by the example of preceding nosologists, than by his own excellent judgment. Of some diseases this famous physician has enumerated as many varieties as there are exciting causes! Upon the whole, we greatly doubt whether such minuteness of diagnosis is often possible, or if it be, whether it is of any avail in actual practice.

We shall now follow the author into his account of the first or acute species. He has never seen any thing that could lead him to suspect dysentery to be contagious. This entirely coincides with our own observations, and not with ours merely, but with that of every modern practitioner with whom we are acquainted. The opinion of Cullen, Pringle, Hunter, Harty, and others, upon this point must therefore be set aside. Either the dysenteries of their day was a different disease from what it now is; or these eminent individuals were betrayed by their preconceived ideas, into a mistake. It is surely of very little present importance which of these alternatives may be the truth; for opinions must now-a-days be decided not by authority, but by the touchstone of facts carefully observed and faithfully recorded.

Mr. Bampfield very candidly admits that he has seldom or never found scybalæ in the stools of dysenteric patients. This is another particular in which his observation coincides with ours. From experience we have long been convinced that these scybalæ either do not exist at all, or at least that they are much rarer occurrences than the older writers would lead us to believe.

The author goes on to describe the symptoms that affect the tongue and fauces, the stomach, intestines and liver, the urinary organs, the vascular and nervous systems, together with the appearances on dissection in dysentery. What he has advanced on these subjects is exceedingly accurate and methodical, but not particularly new. We shall therefore pass this part altogether, as we shall also do the chapters on diagnosis and prognosis, and proceed at once to his observations on the *predisposition* to this *disease.* He is of opinion that the predisposition to an increased secretion from the lining membrane of the organs of smell and respiration in Europe, becomes in India a predisposition to an increased secretion from the villous coat of the intestines. Hence fluxes are as common in the latter climate as coughs and colds are in the former. This conversion or change in the *locale* of increased action, he thinks chiefly attributable to the indulgences in heavy and stimulating diet, and the imprudent exposures to the night air, of which the unwary European newly arrived from his native climate, is wont to be guilty. Atmospheric vicissitudes, by checking perspiration, produce a similar detrimental effect.—We do not recollect that the following circumstance has ever been noticed heretofore as a predisposing cause of dysentery:

"The copious perspiration of the newly arrived European becomes accumulated, when he is sitting or walking, on the lower part of the shirt, more especially about that part of the abdomen where the waist-band of the small-clothes or pantaloons presses against it, the tight or close application of which occasions an increase of heat and of perspiration at this particular part, during the day, and intercepts the exhalation as it flows down the body; hence if he should lie down in this state, cold will be induced on a particular part of the abdomen, by the evaporation of the exhaled fluid from the wet linen in contact with it; perspiration, before profuse, will be now effectually suppressed, and its injurious consequences be felt by the chylopoietic vicera." P. 69.

According to our author's observations, the stools in dysentery are more frequent during the night, and especially towards morning, than at any other period of the four-and-twenty hours. This he seems inclined to ascribe to "solar influence!" We ourselves are thorough sceptics as to planetary influence of any sort on diseases; and we are rather surprised how Mr. Bampfield could yield his powerful understanding to such a notion; more particularly after reasoning so judiciously on *lunar* influence as he does in the following paragraph.

"The periods of dysenteric attacks and relapses I have observed to be more common at the plenilunar and novilunar periods, than at the interlunar intervals. But whether the increased attraction of the moon, at the change and full, has any *direct* power in producing diseases, I believe will never be satisfactorily determined; and notwithstanding the ingenious hypothetical explanations of Dr. Balfour, Dr. Darwin and others, I am induced to conclude that it has only an indirect influence or power by the changes which it occasions at those periods on the atmosphere and winds; for the prevalence of fresh winds, strong gales, and showers of rain, has been observed to be much greater at these periods of the moon, than at the inter-lunar intervals; and these, by checking perspiration, produce effects on the constitution excitive of many acute diseases which have been in part ascribed to the direct agency of lunar attraction on the fluids of the body, by supposing that it decreases the gravity, and diminishes the stimulus, of the particles of the blood." P. 84.

With regard to the proximate cause, our author seems to be of opinion that dysentery is to all intents and purposes inflammation: or if these two diseases are not exactly identical, that, at least the former is attended with analogous symptoms and actions of vessels, and is followed by similar consequences as inflammatory action of other organs of the body. What tends to confirm him in this theory is the disclosure so often made by dissection. On examining the body after death we find visceral enlargements and adhesions, a blood-shot appearance of the intestines, ulcers, abscesses, and sometimes mortification, similar to what are observed after inflammation of other parts, external or internal. These appearances are very striking, yet we hold them to be equivocal. Mr. B. like many others, has been deceived by confounding the ultimate changes with the primary diseased movements. We are, in every case, inclined to regard inflammation rather as a sequence than a consequence of dysentery, as a contingent effect, and not as a uniform result. Indeed the author goes nigh to admit this; for in order to make good his theory he is obliged to extend the term inflammation to *every increased action* of the capillary vessels of secreting membranes. He says,

"Those who do not choose to admit inflammatory action to be, in all cases, the proximate cause of dysentery, in mild and less severe cases, still call it an increased and morbid excretion of the capillary vessels of the intestines, although it is, assuredly, equally philosophical to denominate this action in dysentery inflammatory, as it is the action of the minute secreting vessels of the urethral membrane in gonorrhœa, or of the membranes of the bronchia and nose in catarrh; for in mild cases of those diseases, the pain accompanying them is not constant and acute, nor accompanied with fever, or hard pulse; nor are recoveries often doubtful." P. 90.

All this is very ingenious, but still we are unsatisfied. If dysentery and inflammation be really convertible terms, why (we would venture to ask) are the phænomena of enteritis, and of diarrhœa or dysentery, so radically different in their nature? And, why does not blood-letting ALONE cure fluxes as rapidly and surely as it is wont to do the ordinary forms of true abdominal inflammation? In real idiopathic inflammation of the bowels, obstinate constipation is one of the most characteristic symptoms, owing probably to an almost total suspension of secretion from the villous coat and intestinal glands. Now this suspension of secretion may be fairly attributed to the gorged and obstructed state of the vasa minima, both secernent and exhalent. But, in dysentery, the intestinal secretions instead of being lessened or suspended, are preternaturally increased in quantity, and morbidly altered in quality. Surely such an opposite state of things cannot be attributed to the operation of one and the same cause.

But, although it may be incorrect in speculation to view dysentery and inflammation as one, it will generally be safe in practice to apply to the former the same *principles* of treatment as to the latter. We should never forget that a disease, though not primarily inflammatory, may often have a strong tendency to run into that state. This we believe to be the case in dysentery; consequently we should use the lancet as boldly in the early stage of that disease, as we do in severe cases of spasmodic colic, and with the same views, namely, to remove pain; and (above all) to *prevent inflammation*. Whenever the pulse and heat are high, and the abdomen painful on pressure, that is to say *permanently* painful on pressure, and the pain is confined to any given point, there is reason to fear that local inflammation is begun there; and, thenceforward it behoves us to subdue it by vigorous depletion. The mere intensity of the febrile symptoms, considered *per se*, is by no means to be neglected; for, as the author judiciously observes, "fever rarely exists in the tropics without being occasioned by local inflammation or determination."

This leads us to speak more in detail of the mode of cure laid down by Mr. Bampfield. The remedies he trusts to are 1st. Bleeding; 2d. cathartics; 3d. diaphoretics; and 4th. mercurials. He discusses these under separate heads, and each of them at considerable length. His remarks on blood-letting are singularly valuable, and have our cordial approbation. We think he has deserved well of the profession for the pains he has taken to introduce this remedy to more general attention. It is gratifying to think that experience, on matters of great importance, is always uniform; and that where it finds men willing to obey its dictates, it always conducts them to the same mode of practice. For instance, it was not by any preconcerted opinions that Mr. Bampfield was induced to employ the lancet in dysentery; but by the careful observation of actual cases. We can say the same thing of ourselves, for experience led us to the same conclusions with those here stated by our author. We well recollect the reluctance and trepidation with which we first "wetted a lancet" in a disease where it had been totally proscribed by the concurrent authority of all those authors whose works on the subject were most esteemed. We watched the patient in anxious dread of those formidable consequences which had been alleged to follow venesection. But the result was quite contrary to what we had been taught to expect; for all the severe symptoms were greatly mitigated by the evacuation. Emboldened a little by success, we began cautiously, but regularly, to employ blood-letting whenever the state of the pulse and the heat of skin seemed, on general principles, to warrant it; and ere long we found that dysentery, from being an unmanageable and baffling disease, was converted into a form much more responsible to the ordinary medical treatment. Even when the quantity of blood evacuated by stool was so considerable as to cause debility or prostration of strength, we did not refrain from the lancet: nay we considered the use of it to be rendered if possible, more imperative on that account; for we viewed the hæmorrhage from the intestines to be *active* in its nature, and thought it as incumbent upon us to check it by venesection, as it is to check (by bleeding at the arm) hæmoptysis, or any other internal hæmorrhage. We are convinced that four ounces of blood lost by the anus causes more debility than four and twenty lost by the arm. We look upon blood-letting to be a very great improvement in the modern treatment of dysentery. We give the praise of it to modern times, because, although it was practised and recommended by Sydenham, we greatly doubt whether the limited quantities he was in the habit of taking away, could have exerted any very marked benefit on the disease. It is, we believe, to Dr. Whyte that the profession are indebted for having shewn the perfect safety of this remedy: and had this gentleman lived to publish more extensively upon his experience, we have little doubt that venesection would have been earlier and more effectively adopted in military and naval practice than it has been. But the premature death of this lamented individual, from inoculating himself with the matter of a plague bubo, cut him short in the middle of his honourable career; and the air of rashness which attended the circumstances of his decease, induced many to discountenance the practice (stated in his letter to H. R. H. the Duke of York; see Med. and Phys. Journal, Vol. ii) of bleeding to syncope in dysentery, as the hazardous experiment of a well-meaning, but hot-headed medical enthusiast. In consequence of this prejudice, blood-letting never became fully established as a remedy in this disease until the late Peninsular campaigns. Experience there pointed out to military medical gentlemen a similar mode of treatment to what had suggested itself to Mr. Bampfield and others of his naval brethren, employed within the tropics. The whole of our author's section on this subject is so excellent, that we are at a loss what paragraph to extract in preference to another. We take the following passages almost at random.

"In dysentery it happens that a certain degree of debility must be induced either by the antiphlogistic regimen, or by the protracted disease gradually exhausting the animal and vital powers; hence it is thought preferable to induce a certain degree of it at once, (by bleeding, to wit) and thus put a speedy termination to the disorder, and prevent the distressing, and sometimes fatal effects of the chronic stage."

"In this disease, venesection is said to be injurious by Dr. J. Clark, (p. 324, 325) and probably his authority has given rise to the neglect and omission of the practice. He admits that "no evacuation is better calculated for the relief of the patient, when the disease is accompanied with a fever of the inflammatory kind. But, in hot climates, fluxes being either of a chronic nature, or accompanied with a low fever, the strength of the patient sinks from the beginning."

It is granted that there is a peculiar sensation of debility, the companion of the very severe and inflammatory varieties of dysentery, resembling what occurs in enteritis, and this sensation is maintained and increased by the constant dysenteric evacuations, the severe pains, the want of sleep, and the exhaustion of the sensorial power in the sensitive and irritative motions: but as no judicious practitioner is deterred from bleeding by the peculiar sensation of debility attending gastritis and enteritis, so let no one be deterred from employing it in the inflammatory forms of dysentery. It has been already remarked, that the chronic stage is generally a sequela to the severe and inflammatory varieties, if their acute stage be not arrested and cured. If bleeding be not employed in the inflammatory variety, either death, or a very long chronic stage, almost invariably ensues. Hence, bleeding often does away with the "chronic nature of fluxes." I have not observed that the "fever" which accompanies dysentery is particularly "low"; however, Dr. Cullen, in his *Nosology*, has enumerated "typhus fever," as a characteristic symptom of enteritis, but he nevertheless recommends bleeding for its cure. The author adds—

"Venesection can be dispensed with in the milder and safer forms of dysentery, where the symptoms of inflammation are not present, where the pain is only occasional, and the evacuations are not copious nor frequent: these varieties will, in general, yield to the other remedies employed for the cure of dysentery." P. 110, 111, 113.

We cannot dismiss this important topic without making one more extract. It relates to Dr. Maclean, who has lately earned so much notoriety by his "Researches" on Plague and Epidemic Diseases. The opinions of this gentleman are so exceedingly crude and hypothetical, that their refutation might safely be entrusted to any young gentleman who has attended a course or two of lectures, and walked the hospitals for a single season. But while they are deemed of importance enough to sway the East India board, and to claim the deliberations of a parliamentary committee, we feel a sort of obligation to notice them, however little they may intrinsically deserve such a compliment.

"Since this work has been in the press, (says Mr. Bampfield,) I have been astonished and shocked to find blood-letting in hot climates condemned 'as *a pernicious error*;' as a 'deleterious practice,' and as '*an evacuation injurious in all diseases*,' by Dr. C. Maclean, Lecturer on Diseases of Hot Climates to the Hon. East India Company, who declares 'that blood-letting is an operation which ought never to be performed under any circumstances, or in any situation;' and that his 'observations were almost all written *a*

*great many years ago,* in the East Indies!' *See 'Results of an Investigation,' &c. Vol.* ii. *Preface* 11.—Surely, with these sentiments, Dr. C. Maclean cannot have tried it; and, with every deference, I submit to him the *results* of the cases annexed to this work. As Dr. Maclean has been in Egypt, I would refer him to lib. ii, cap. 7, Prosperi Alpini de Medicina Ægyptiorum, in which he will find that 'Alexander Trallianus, Ætius, atque *alii multi gravissimi medici,*' were of opinion with him, 'optimum esse remedium dysentericis, modicam sanguinis evacuationem.' He also argues with me, that 'si non mittatur sanguis, atque eo modo statim in principio divertatur fluxus ab intestinis,' much more blood and strength will be expended, 'perseverante illo affectu,' than if bleeding be employed. He also mentions two cases cured by bleeding alone, 'sola sanguinis missione.'" P. 114—115.

In fact there is no lack of authorities on this point. Mr. Bampfield might have also quoted in his favour, Galen, Riverius, Amatus the Portuguese, Botallus, Physician to the French Embassy at the Court of Queen Elizabeth, White, ("*de rectâ sanguinis missione.*" London, 1712.) Heister, and Dr. Donald Monro—all strenuous advocates for venesection in dysentery.

We cannot bestow so much commendation upon our author's chapter on cathartics as upon that on bleeding. We conceive the purgatives recommended by him to be far too drastic and stimulating; and we entertain very serious doubts whether jalap, extract of colocynth, or infusion of senna, can be with propriety employed in any stage of dysentery. Surely, on his own notions as to the strictly inflammatory nature of the disease, these medicines must be highly unsuitable; for would it not follow, from his doctrine, that they should aggravate the symptoms? What practitioner will venture to prescribe drastic purgatives in enteritis, or to excite vehement action in an intestine whose calibre is already inflamed? If it is necessary to give rest to an inflamed muscle, or to withhold the stimulus of light from an irritable eye, it is no less necessary to tranquillize and soothe the bowels by all the means in our power. In dysentery, when purgatives are necessary, (and generally they are indispensable), we never employ any other than those of a mild and lubricating nature. Castor oil is almost the only one that is proper; and when it is necessary to increase its activity, that can be readily accomplished by adding to it a few grains of calomel. Indeed Mr. B. himself is fully aware of the virtues of this medicine. The following passage is an excellent one, though rather at variance with his recommendation of the dry and more acrid purgatives.

"The oleum ricini is perhaps better calculated to afford relief in dysentery, than any other aperient or cathartic; for its action is not only mild and generally effectual, but I have observed that some of it passes undecomposed, in its oily form, through the intestines, and appears on the surface of the excrement, and hence may serve as a sort of sheather or defence to the diseased intestines from the stimulus of fæces and morbid secretions." P. 124.

The observations on diaphoretics contain nothing new; we shall therefore pass on to the subject of mercurials.

The author has been in the habit of prescribing calomel, but he seldom gives it alone. He thinks it greatly better to combine it with other purgatives, or with ipecacuanha. This remedy is generally given with the view of correcting the condition of the liver; for all practitioners concur in thinking that the function of this mighty gland is greatly depraved in dysentery, though they may differ in opinion as to the relative importance of this depraved state—some regarding it as the primary cause of the symptoms; and others viewing it merely as one link in the chain of effects. It would probably be alike tiresome and unprofitable to our readers were we, in this place, to enter into minute discussions on the subject. We shall therefore waive the matter altogether, only remarking that we suspect the liver has not, till lately, been allowed its due share of importance among the phenomena of this disease. We are persuaded that much of the exquisite pain and tormina is assignable to vitiated bile passing over the irritable, excoriated, or ulcerated surface of the intestines; for we do not see how otherwise the pain, which succeeds the fullest operation of a cathartic, is to be accounted for. The renal discharges also afford an additional presumption that unhealthy bile performs an important part in the malady. When the urine is collected, it is generally of a green or yellowish colour, and tinges linen, evidently from the admixture of bile; and it is generally passed with considerable heat and smarting. The latter uncomfortable sensation is always ascribed to *sympathy* betwixt the rectum and bladder; but instead of taking for granted that tenesmus is the cause of the difficult micturition, it is more reasonable to believe that the bile, mixed with urine, is the occasion of that teasing phenomenon.

The mercurial preparations prescribed in dysentery are found to produce a solution of the disease; but whether they do so by rectifying the hepatic secretion, or by producing some more secret and inexplicable change in the system at large, is, at present, quite unknown. One thing, however, is certain :—as soon as ptyalism takes place, the disease generally disappears as a matter of course.

In consequence of this last fact being so universally noticed, some practitioners have directed their views to salivation as the sole indication of cure, and have boldly prescribed calomel alone in doses of one scruple twice or thrice a day. This we hold to be a great improvement in tropical practice; it was extensively used by Dr. Johnson, in India, and by him communicated to the profession in his various writings. We ourselves have employed the scruple doses in the dysentery of the western hemisphere, and have seen it, in the great majority of instances, produce all the benefit which Dr. Johnson taught us to expect. It deserves to be remarked, however, that it is a practice only adapted to tropical climates, for *there* the human frame is much less susceptible of the action of mercury, and consequently will bear much larger doses of that metal than it would be prudent to prescribe in the climate of this country.

Mr. Bampfield appears to undervalue this mode of treatment, for various reasons; these we shall state in his own words. In a note at P. 141, he observes—

"Scruple doses of calomel are recommended 'two, three, or four times a-day,' in Dr. Johnson's valuable work on the Influence of Tropical Climates, P. 213; but such large doses appear to me to be rarely necessary, and in general not proper; for if they be intended as a purgative, I would observe there are others less uncertain, more safe, and that do not increase the hepatic secretions so very copiously, see page 117; and if to excite salivation in a very short period, I would urge that such an object does not in general seem necessary, and the attempt in some cases is improper; for in the inflammatory cases, fraught with danger, the induction of salivation is incompatible with a high degree of inflammation, P. 136; and when that is subdued, the absence of immediate danger enables us to proceed leisurely to effect it, if the subsequent symptoms require it, P. 138. Besides, these doses may salivate profusely as well as speedily, to which objections are offered in the paragraph above, and which particularly apply to the cases of British sailors at sea, among whom the practice originated, and among whom I have witnessed fatal terminations for want of a proper diet: and salivation is not in all cases necessary. If they be intended to excite a mercurial fever or irritation in the system speedily, such a result has appeared to hasten the catastrophe, if affected *(effected)* before high inflammation is subdued."

When we consider that the remarks we are now making are destined to appear in Dr. Johnson's own Journal, we feel a little at a loss, from motives of delicacy, how to proceed in a matter so personal to himself. Nevertheless, since speak we must, let us speak impartially. "Amicus Socrates—amicus Plato,—*sed magis amica veritas*": we shall, therefore, endeavour to decide as uprightly as if we were utter strangers to the talents of the two respected individuals who differ on the point now before us.*

In the first place, we put it to Mr. Bampfield's own candour, whether it is not somewhat uncourteous never to allude (except for the purpose of finding fault) to Dr. Johnson's work on Tropical Climates, a work which has justly acquired a high degree of public esteem. Surely he is paying a bad compliment to a publication of such ability, when he huddles the only remarks he makes upon it into a foot-note! In the second place, the practice of scruple doses did not "originate among British sailors," but was first tried by Dr. Johnson *on himself*. Now, this effectually does away with any unpleasant impression which might possibly be excited by imagining, that this new practice had been first tried as an experiment upon the untutored and unfriended seaman. Thirdly, when Mr. B. quotes Dr. J's recommendation of the scruple doses, "twice, thrice, or four times a-day," he should also have quoted another sentence in the paragraph, which operates as a complete saving-clause to the former. It is the following: "I did not indeed, adopt this practice generally, being quite satisfied in ordinary circumstances with the plan which I have above detailed."—*Dr. Johnson on Trop. Clim. 2d Edit. P.* 214.

But it is humiliating to discuss such personalities; we shall, therefore, pass them over, and argue the point upon general grounds.

We frankly admit, that the first stage of dysentery cannot be treated on principles too strictly antiphlogistic; but we contend, that when the second stage has commenced, or, in other words, when the previous increased action has ended in congestion, nothing can be more useful than to saturate the system with mercury.

---

* Aware of this clash of sentiment, the Editor sent Mr. Bampfield's book to a Gentleman of extensive experience, and unrivalled talents, a friend also of Mr. Bampfield's, requesting him, as indeed he does upon all similar occasions, to speak freely and candidly, and not to spare either party, wherever he disagreed in opinion with them. For the truth of this statement, Mr. Bampfield himself can vouch.

This mineral does more to resolve irritative fever, to equalize the circulation, disgorge the capillary vessels, restore the balance of the nervous power, and open the sluices of the various healthy secretions and excretions, than any other remedy with which we are acquainted. Besides, it should be remembered, that calomel is a restringent as well as a cholagogue, and that its efficacy consists as much in restraining and rectifying the biliary and intestinal secretions, when they are excessive or morbid, as in exciting and augmenting them when they happen to be torpid or too scanty.

The propriety of impregnating the constitution, then, being admitted, the only question of importance is, how is it to be done most speedily? We answer with confidence, "By means of calomel in scruple doses, night and morning." We should recollect, that the cases to which alone this practice is applicable, are pregnant with great distress and danger, and that, consequently, delays are dangerous. Nothing but the most energetic practice will prove available to save life, and *that* even, in too many instances, fails. Upon the whole, deferring to Mr. Bampfield's judgment and experience, but at the same time abiding by our own, we must take the liberty to declare, that we consider all his fears about excessive salivation, hypercatharsis, and so forth, as the results of this new practice, to be entirely illusory. His opinion that "the induction of salivation is incompatible with a high degree of inflammation," not only takes for granted the correctness of his own theory of dysentery, but is in itself perhaps little better than a hypothesis. Besides, it carries no weight with it as an objection; because, where is the practitioner that would proceed to mercuralize the system until he has reduced the existing febrile excitement? Neither we ourselves, nor (we apprehend) Dr. Johnson, have ever administered scruple doses, or any other doses of calomel, with an attempt to salivate, without premising active depletion both by blood-letting and purgatives.

These discussions have grown under our hands insensibly, and almost involuntarily, to a length that has filled our limits, and will compel us to hasten precipitately to a conclusion. This we regret the more, because we must pass over in unmerited silence, crowds of important observations on chronic and scorbutic dysentery. The author's delineation and treatment of these varieties is no less able than his delineation and treatment of the acute species. His chapter on chronic dysentery, is chiefly valuable on account of the clearness and earnestness with which he points out the necessity of dietetic restrictions as auxiliary to the medicines employed. He details several very illustrative cases where irregularities, whether in eating or drinking, brought on fatal relapses.

"The evil and mortal consequences resulting from intemperance, imprudent indulgences of the appetite, and of the social disposition, have been depicted in treating of the variety A: these errors are more pernicious in this, and the necessity of a most regular and temperate life, and of a strict dietetic regimen is consequently greater. Obstinate or ill-fated patients, are sometimes met with, who cannot be persuaded, or induced by sufferings, to a proper diet. I have sometimes eluded the bad effects of their folly and obstinacy, by keeping up a slight mercurial soreness of mouth, which has compelled them to relinquish solid food, and to live on broths and farinaceous preparations of diet, so long as to allow of a favourable state of quiescence to the bowels, and to admit of the establishment of a healthy action of the ulcer in the intestines. We have no indirect means of adroitly warding off the fate of the determined inebriate, and can only succeed by resolute compulsion." P. 242.

Perhaps the most original portion of the volume is the part that treats of scorbutic dysentery. We read it with very great pleasure, and give Mr. Bampfield the highest praise for the number of curious, instructive, and interesting facts which he has collected on the subject of scurvy. Yet we have our doubts as to the correctness of his nomenclature, when he speaks of *scorbutic dysentery*: we indeed suspect that it ought rather to be considered an accidental co-existence of the two diseases in the same subject, than a distinct and specific variety of dysentery. This, however, is in a great measure, matter of opinion.

He relates some singular cases, where scurvy appeared in the men in a week, or less, after putting to sea; and others, where the sea air was the only obvious cause. The pathology of scurvy is still very obscure, notwithstanding all the experience of the late war; that neither salt meat, sea-air, nor atmospheric heat is indispensably necessary to the production of the disease, is proved by what Dr. Gregory relates of a family that came under his observation, who suffered severely from scurvy, during a season of dearth, in consequence of their chief diet having been tea. They had used it three times a day.

Before closing Mr. B's valuable volume, we shall glance at the preface, which in this as in other books, though the first in order, is always the part last written. In it the author makes some remarks on a paper annexed to the 2nd Edition of Dr. Johnson's Work on Tropical Climates, by Archibald Robertson, M.D. The subject is the Medical Topography of New Orleans, and the diseases (chiefly dysentery), which affected the fleet and army during the ill-fated expedition against that place, in 1814, 15. In the fatal cases Dr. Robertson found no morbid intestinal appearances sufficient to account for death, but *always* extensive structural derangement of the liver, and generally abscess of that organ. Mr. Bampfield tries to explain these appearances by the cold climate of New Orleans during the winter, and the rapid transition from the hot climate of Jamaica, to the cold one of New Orleans, which the crew sustained. These causes united, he thinks, were very likely to produce acute inflammation of some of the abdominal viscera. We suspect this explanation will not do, because we happen to know (and we have the information from Dr. Robertson himself) that almost all the fatal cases occurred in March, April, and May, when the cold season at New Orleans had gone by, and the thermometer had permanently risen to the average standard of the torrid zone. Indeed, Dr. R. particularly observes, that this form of dysentery, unlike that of cold climates, was aggravated, instead of being mitigated by high atmospheric temperature. Even Mr. B. himself admits, in the face of his explanation, that "these peculiarities cannot be very satisfactorily explained." For our part we are clearly of opinion that they cannot be explained at all, unless we admit what Dr. R. labours to establish, namely, that many cases of tropical dysentery are nothing more than hepatic flux: and, consequently, that the liver is the hidden but real source of all the dysenteric symptoms; a conclusion embraced by Mr. Ballingall at p. 80 and 91 of his late work.

This last opinion was advanced and maintained at considerable length, by Dr. Ferguson, Inspector of Hospitals, in an able paper in the Medico-Chir. Transactions, vol. ii. Dr. Robertson, from the light of symptoms before, and dissections after death, was led to similar conclusions; and took up the very same ground, without knowing that it had been pre-occupied by Dr. Ferguson, or indeed by any one else. For a detail of the circumstances that led him to adopt these views, we must refer to his paper, in Dr. Johnson's work, before mentioned. He has, as might be expected, acquired a bias to the hepatic theory of dysentery; a theory which, we need hardly observe, is quite irreconcileable with that of Mr. Bampfield.—But we forget; it is not our business to dogmatize about probabilities, or to attempt settling the merits of rival hypotheses.

Notwithstanding our objections to some of Mr. B.'s doctrines, we entertain a high opinion of his work. The talent, learning, and sagacity it displays will render it a rich treat to those who are fond of a well-written and well digested treatise on this fatal disease. To them we conclude by recommending it.*

---

* The Editor begs leave to append a remark or two to the above able Review of Mr. Bampfield's work. Sixteen years ago, viz. in the month of Oct. 1803, while suffering under a severe attack of what Mr. B. denominates *Dysenteria Inflammatoria*, at Bengal, the Editor was bled, as a matter of course, by his friend Mr. Rogers, Surgeon of the Preston Indiaman, then in the Hoogly, and the practice was pursued by himself and others afterwards, as laid down in his work on tropical climates. Mr. White, a navy surgeon, in the year 1712, wrote a book on this very subject, wherein he lays down a system of blood-letting in dysentery, far more efficient than any modern author—not even excepting Mr. Bampfield. This work the Editor gave to his esteemed friend Mr. Hennen, some years ago, and is noticed at page 48 of Mr. H's excellent work on Military Surgery. All idea of *novelty*, therefore, in the practice, is quite out of the question. That dysentery and *inflammation* are not identified by Mr. Bampfield's arguments, is pretty clear from this circumstance, that he applies the character *inflammatory* to only *one* variety, excluding it from the "*acute*" and "*severe*" forms of the disease!

That inflammation of the *mucous* membrane of the intestines is not always, or even generally accompanied by dysenteric symptoms, is proved by Broussais, who finds, or says he finds, *inflammation* there as the cause of *all* fevers. It will be pretty readily granted, that *all* fevers are not characterised by dysentery, but rather by an opposite state of the bowels. Broussais or Bampfield, therefore, must be wrong—perhaps they are both a little astray.

